# Effects of nanoselenium on the performance, blood indices, and milk metabolites of dairy cows during the peak lactation period

**DOI:** 10.3389/fvets.2024.1418165

**Published:** 2024-06-20

**Authors:** Ming Xiao, Yajing Wang, Manlin Wei, Wen Peng, Yuxiang Wang, Runze Zhang, Yongjie Zheng, Ji Ju, Chenyang Dong, Liu Du, Meili Bao

**Affiliations:** ^1^College of Animal Science and Technology, Inner Mongolia Minzu University, Tongliao, China; ^2^College of Animal Science and Technology, China Agricultural University, Beijing, China

**Keywords:** cow, nanoselenium, blood indicators, blood selenium, milk selenium, milk metabolites

## Abstract

To compare the impact of nanoselenium and sodium selenite on the performance, blood indices, and milk metabolites of dairy cows during the peak lactation period, two groups of dairy cows under the same conditions were selected as the control group (CON group) and treatment group (NSe group) for a 38-day (10 days for adaptation and 28 days for sampling) experiment. The control group (CON) was provided a basal diet +3.3 g/d of sodium selenite (purity1%), whereas the nanoselenium group (NSe) was offered the same diet +10 mL/d of nanoselenium (selenium concentration 1,500 mg/L). The results showed that NSe significantly increased the milk yield, milk selenium content, and feed efficiency (*p* < 0.05), but had no significant effect on other milk components (*p* > 0.05). NSe significantly increased blood urea nitrogen (BUN) and alkaline phosphatase (ALP) (*p* < 0.05), but had no significant effects on malondialdehyde (MDA), superoxide dismutase (SOD), glutathione peroxidase (GSH-Px), blood total antioxidant capacity (T-AOC), or blood selenium (*p* > 0.05). In addition, the nontargeted metabolomics of the milk was determined by LC–MS technology, and the differentially abundant metabolites and their enrichment pathways were screened. According to these findings, NSe considerably increased the contents of cetylmannoside, undecylenoic acid, 3-hydroxypentadecanoic acid, 16-hydroxypentadecanoic acid, threonic acid, etc., but decreased the contents of galactaric acid, mesaconic acid, CDP-glucose etc. Furthermore, the enriched metabolic pathways that were screened with an impact value greater than 0.1 included metabolism of niacin and niacinamide, pyruvate, citrate cycle, riboflavin, glycerophospholipid, butanoate and tyrosine. Pearson correlation analysis also revealed a relationship between different milk metabolites and blood selenium, as well as between milk selenium and blood biochemical indices. In conclusion, compared with sodium selenite, nanoselenium improves the milk yield, feed efficiency, and milk selenium content of dairy cows and regulates milk metabolites and related metabolic pathways in Holstein dairy cows during the peak lactation period, which has certain application prospects in dairy production.

## 1 Introduction

Selenium (Se), an important trace element for animals, is extensively involved in physiological metabolic processes and is of the utmost importance for reproductive functions, animal growth, and immunity (1, 2). Selenium exists in various forms in animals, but mainly as selenoproteins, including glutathione peroxidase (GSH-Px), thyroxine deiodinase (DIO), and thioredoxin reductase (TrxR) (3). Because selenium is often closely related to the immune activity and reproductive performance of animals (4), selenium deficiency can lead to many diseases, including oxidative stress, cardiomyopathy, and placenta retention, in animals. However, excessive amounts can also lead to chronic or acute selenium poisoning (5, 6), resulting in reduced production performance.

Selenium in nature usually exists in two forms: inorganic and organic (7). For a long time, sodium selenite has been a commonly used dietary supplement, but its utilization has become limited due to its low bioavailability, ease of overdose, strong toxicity, and environmental pollution (8). In comparison, organic selenium is considered to possess high efficiency, low toxicity and pollution, a high absorption rate (9), and safety (10). To ensure the effective intake of selenium, overaddition is usually adopted in animal production. Due to the relative proximity of the required and toxic doses, both inorganic selenium and organic selenium can easily cause poisoning. The type of selenium has a great influence on its toxicity: selenides are highly toxic, and elemental selenium has low toxicity because it is not readily absorbed by animals. However, nanoselenium is much safer because its toxicity is between elemental selenium and organic selenium (11). From a chemical point of view, nanoselenium is red elemental selenium (zero valence) with a particle size of 20–60 nm that is dispersed around the protein (12). However, the absorption and utilization capacity of nanoselenium in the small intestine may be enhanced by its unique chemical structure and nanoscale effects (13). This may be attributed to its beneficial effects on the duodenum, as evidenced by an increase in length, as well as on the ileum and jejunum, as measured by an increase in villus height, surface area, and goblet cell density (14). Studies have shown that nanoselenium can reduce or prevent oxidative stress (15); it also has antiviral effects or can enhance the effectiveness of vaccination. Moreover, the acute and subacute toxicity of nanoselenium is much lower than that of inorganic selenium (16). In contrast, nanoselenium can alleviate cadmium-induced liver fibrosis in chickens (17), interferes with cadmium-chlorine-induced sperm malformation, and has antiviral effects (18). Studies in production animals also revealed improvements in the growth performance, carcass composition, and immune function of broilers supplemented with nanoselenium (19, 20), which can also regulate the rumen fermentation of sheep and improve the health of goats (21, 22). Moreover, it has the potential to increase the expression of selenium-containing enzymes in the mammary gland of dairy cows and GSH-Px activity in the blood (23). Therefore, nanoselenium has certain applications.

There are more than 40 countries in the world with varying degrees of Se deficiency in soil (24). At present, the soil in most provinces of China is also at risk of selenium deficiency (0.125 ≤ Se < 0.175 mg/kg), and the blood selenium content of the population in most provinces is close to the lower limit of clinical nutritional status (100–200 μg/L) when additional supplementation is excluded (25). Compared with consuming selenium-supplementing drugs, people prefer to obtain selenium through food. The intake of selenium-rich food or diet is helpful for improving or solving the nutritional and health problems caused by selenium deficiency in humans and animals (26). In recent years, various selenium-rich foods, such as selenium-enriched milk, tea, rice, and eggs, have been favored by people (27), and methods of supplementing selenium in animal feed to produce selenium-rich products are valued by practitioners.

Milk products are the most common foods and are a good source of minerals. Studies have shown that increasing the selenium content in milk is a feasible way to meet people’s selenium requirements (28). Dietary selenium can be secreted into milk to obtain selenium-rich milk (29). However, as a new type of selenium supplement, nanoselenium has rarely been studied in dairy cows, especially the efficiency of nanoselenium transfer from feed to the blood and milk of animals, and the metabolites in milk also need more research. Therefore, the effects of nanoselenium on the performance, blood biochemical indices, selenium levels and milk metabolites of dairy cows during the peak lactation period were studied. This study provides a reference for the application of a safe and efficient selenium supplement in dairy production.

## 2 Materials and methods

### 2.1 Experimental design and animal feeding management

Two groups of healthy Holstein cows with similar body weights, parities, and lactation periods of 45–60 days were selected as the control group (CON group) and the treatment group (NSe group), with 339 and 185 cows, respectively, for the 38-day (10-day adaptation and 28-day measurement) experiment. The basal diet was formulated based on the Nutrient Requirements of Dairy Cattle (30) (Table 1). The CON group was given a basal diet +3.3 g/d of sodium selenite (purity1%), whereas the NSe group was given a basal diet +10 mL/d of nanoselenium (selenium concentration 1,500 mg/L). Sodium selenite and nanoselenium were premixed with corn meal and then mixed into TMR diets supplemented with other feed materials. The selenium content in the diets of both groups was 15 mg per day. Cows were fed twice (07:00 and 15:00), milked three times (07:00, 15:00, and 23:00) every day, and had free access to drinking water. The milk yield and feed intake were recorded daily and clinical indications of selenium poisoning were evaluated (Annex 1 for evaluation criteria). At the end of the trial, milk samples from 27 cows in each group were randomly collected to analyze the milk composition, while blood samples from 10 cows in each group were collected randomly to determine the biochemical indices, and 6 samples from each group were used to determine the selenium content in the blood and milk.

**Table 1:** TMR diet composition and nutrient level (dry matter basic).

Ingredients	Content (%)	Nutrient levels	Content (%)
Alfalfa	15.04	Net lactation energy NEL/ (MJ/kg)^2^	7.32
Corn silage	23.13	Crude protein	18.11
Flaked corn	16.65	Ether extract	3.00
Corn	2.32	Crude ash	6.42
Soybean hulls	11.30	NDF	28.84
Soybean meal	8.33	ADF	25.69
Low fat DDGS	8.79	Ca	0.98
Corn germ meal	2.94	P	0.38
Mildew remover	0.10		
Corn gluten meal	4.60		
Urea	0.21		
Glucose	1.22		
Fat powder	1.80		
Rumen buffer (sodium bicarbonate and magnesium oxide 2:1)	0.83		
Yeast	0.10		
Mycotoxin-binding agents	0.10		
Premix^1^	2.54		
Total	100		

^1^Each kg of premix contained vitamins A (800 000 IU), D3 (180,000 IU), E (15,000 IU), B1 (920 mg), B2 (1,200 mg), and B12 (10 mg), niacin (30,000 mg), iron (1,000 mg), copper (680 mg), manganese (350 mg), zinc (800 mg), magnesium (198,400 mg), and cobalt (20 mg). ^2^The net energy of lactation is a calculated value, and the others are measured values.

### 2.2 Feed intake record and feed sample analysis

The total and residual amount of the TMR diet of each group were recorded daily. Representative TMR diets were sampled every day and mixed in equal amounts for nutrient analysis (moisture monitoring daily). After being dried at 65°C to a consistent weight and crushed, the samples were passed through a 1 mm sieve. Dry matter (DM) was assessed by drying to a constant weight at 105°C and used to calculate dry matter intake (DMI) on the basis of individual feed intake. AOAC (2005) methods were utilized to analyze the feed components, including crude protein (CP), crude ash (Ash), and ether extract (EE) (31). Van Soest’s method were employed to examine the neutral detergent fiber (NDF) and acid detergent fiber (ADF) contents (32).

### 2.3 Milk yield and milk composition analysis

During the experimental period, the daily milk yield was recorded and 4% milk fat corrected milk (FCM) yield was calculated. The composition of fat, protein, lactose, and ash in the mixed milk samples (morning, middle, and evening milk = 4:3:3) of 27 cows in each group was determined using a LM2 milk composition analyzer (Harrod Beijing Technology Co., Ltd.) at the end of the trial.

### 2.4 Blood sampling and analysis

At the end of the experiment, blood samples from 10 cows in each group were collected randomly to determine the biochemical indices. Blood (10 mL) was sampled from the tail root vein 1 h before the morning feeding, allowed to stand at 4°C for 4 h, and then centrifuged (3,000 r/min, 10 min). Serum was collected to determine biochemical indices, antioxidant indices, and selenium contents.

The blood antioxidant indices superoxide malondialdehyde (MDA), superoxide dismutase (SOD), glutathione peroxidase (GSH-Px), and total antioxidant capacity (T-AOC) were determined using biochemical detection kits (Nanjing Jiancheng Bioengineering Institute, China) on an enzyme labeling instrument (Huawei Delang DR-200BS, Wuxi, China). Furthermore, the levels of glucose (GLU), creatinine (CRE), urea nitrogen (BUN), alanine aminotransferase (ALT), lactate dehydrogenase (LDH), aspartate aminotransferase (AST), γ-glutamyl aminotransferase (γ-GT), alkaline phosphatase (ALP), and cholinesterase (CHE) were also measured with A6 semi-automatic biochemistry analyzer (Beijing Matsushige Technology, Beijing, China).

### 2.5 The content of selenium in blood and milk

Blood and milk samples from 6 cows in each group were randomly collected to determine the selenium content via 3,3′-diaminobenzidine (DAB) spectrophotometric colorimetry. In the blank and standard tubes, 100 μL of the standard material and distilled water were added, respectively. Acetic acid buffer (400 μL) was added simultaneously to the blank, standard, and sample tubes, followed by the addition of 200 μL of EDTA-NA2 solution. 200 μL of color-developing solution was then added to the sample tube, followed by thorough mixing of all the components. In the dark, 400 microliters of alkaline solution were added to a water bath at 60°C for 20 min. After shaking, the mixture was left for 5 min, 200 microliters were removed, and the solution was placed on an enzyme marker plate to determine the optical density (OD).

### 2.6 Nontargeted metabolomics analysis

Nontargeted metabolomics of the milk was determined by LC–MS, and the differentially abundant metabolites and their enrichment pathways were screened. Once the milk sample had naturally thawed at room temperature, 200 μL of milk sample was transferred to a 1.5 mL centrifuge tube. Then, 400 μL of an equal volume of an extraction solvent composed of acetonitrile and methanol was introduced. The sample was vortexed for 30 s and extracted by ultrasonication at a low temperature for 30 min (5°C, 40 kHz). After exposing the sample to −20°C for 30 min, it was centrifuged at 4°C for 10 min at a speed of 13,000 rpm. Then, the supernatant was discarded, and the sediment was redissolved in 100 μL of complex solution (water: acetonitrile = 1:1). Subsequently, the solution was extracted using ultrasonication at a low temperature (5°C) for 5 min at 40 kHz and centrifuged at the same speed at 4°C for 5 min. Using internal intubation, the supernatant was poured into a vial with an internal cannula to prepare for machine analysis. Detection was conducted by ultrahigh-performance liquid chromatography-tandem Fourier transform mass spectrometry (UPLC-FT-MS) on an LC–MS platform (UHPLC-Q Exactive HF-X system). The machine conditions utilized included a chromatographic column (ACQUITY UPLC HSS T3, 100 mm × 2.1 mm i.d., 1.8 μm; Waters, Milford, USA), and the column temperature was 40°C; mobile phases A and B were composed of water (95%) and acetonitrile (5%) with formic acid (0.1%) and acetonitrile (47.5%), isopropyl alcohol (47.5%), and water (5%) with formic acid (0.1%), respectively. The sample volume was 3 μL, and the column temperature was 40°C. Mass spectrum conditions: electrospray ionization and negative and positive ion scanning modes for mass spectrum signal collection, respectively.

### 2.7 Statistical analysis

The production performance, blood index, and milk content data were entered into Excel (2016) and analyzed by Student’s *t*-test using JMP13.0 software (SAS Institute, Japan). The results are presented as the standard errors and mean values. Differences were considered significant and extremely significant when *p* < 0.05 and *p* < 0.01, respectively. In addition, a difference analysis of the matrix files after metabolomics data preprocessing was carried out. The stability of the model was assessed through 7-cycle interactive validation, and partial least squares discriminant analysis (PLS-DA) was adopted using the R software package ropls (version 1.6.2). Multiple difference analyses and Student’s *t*-test were also conducted. The *p* value and the variable influence on the projection (VIP) of the partial least squares discriminant analysis (PLS-DA) model were utilized to evaluate the differentially abundant metabolites. When VIP > 1, *p* < 0.05, and FC > 1 or FC < 1, the differentially abundant metabolites were identified from the KEGG database^1^ and involved in the pathways that were analyzed by the Python software package SciPy. Fisher’s exact test was used to determine the statistical data and the most relevant biological pathways. Pearson correlation analysis was conducted among the milk metabolites, the blood selenium content, the milk selenium content and blood biochemical indices.

## 3 Results

### 3.1 DMI, milk yield, and milk composition

As shown in Table 2, the milk yield and feed efficiency significantly increased in the NSe group compared with those in the CON group (*p* < 0.05). However, the DMI, FCM yields and the protein, fat, ash and lactose contents of the milk did not differ significantly (*p* > 0.05). Moreover, no indication of poisoning was observed during the experiment.

**Table 2:** Effects of nanoselenium on the DMI, milk yield, and milk composition of dairy cows during the peak lactation period.

Items	CON	NSe	SEM	*p*-value
DMI (kg/d)	20.78	20.11	0.25	0.0667
Individual milk yield (kg/d)	23.02B	24.63A	0.16	0.0001
4% FCM (kg/d)^1^	22.46	23.63	0.41	0.0521
Feed efficiency^2^	1.09b	1.18a	0.02	0.0107
Milk composition (%)				
Fat	3.84	3.73	0.43	0.8253
Protein	3.58	3.58	0.03	0.9627
Lactose	5.27	5.26	0.05	0.9507
Ash	0.78	0.78	0.01	0.9165

Different lowercase or capital letters on the shoulder indicate a significant (*p* < 0.05) or an extremely significant difference (*p* < 0.01), respectively. The same or no letters indicate no significant difference (*p* > 0.05). The following tables are the same. ^1^4% FCM (kg/d) = 0.4× individual milk yield (kg/d) + 15× individual milk fat yield (kg/d); ^2^Feed efficiency = 4% FCM (kg/d)/DMI (kg/d).

### 3.2 Serum antioxidant indices

Table 3 shows that nanoselenium had no statistically significant impact on the serum levels of GSH-Px, T-AOC, SOD, or MDA in dairy cows (*p* > 0.05).

**Table 3:** Effects of nanoselenium on the serum antioxidant indices of dairy cows during the peak lactation period.

Items	CON	NSe	SEM	*p*-value
Glutathione peroxidase (U/mL)	1137.53	1145.92	19.73	0.7670
Superoxide dismutase (U/mL)	99.20	98.20	2.37	0.7700
Total antioxidant capacity (U/mL)	8.94	8.87	0.22	0.8436
Malondialdehyde (U/mL)	5.02	5.19	0.21	0.5834

GSH-Px, glutathione peroxidase; T-AOC, total antioxidant capacity; SOD, superoxide dismutase; MDA, malondialdehyde.

### 3.3 Serum biochemical indices

As shown in Table 4, compared to CON group, NSe significantly increased the serum BUN and ALP levels (*p* < 0.05) but had no significant impact on other serum biochemical indices (*p* > 0.05).

**Table 4:** Effects of nanoselenium on the serum biochemical indices of dairy cows during the peak lactation period.

Items	CON	NSe	SEM	*p*-value
ALT (U/L)	36.69	37.18	1.93	0.8597
AST (U/L)	62.93	62.45	2.28	0.8846
BUN (mmol/L)	2.08b	3.28a	0.08	0.0001
GLU (mmol/L)	4.49	4.55	0.05	0.4097
CRE (umol/L)	132.51	137.69	2.53	0.1655
ALP (U/L)	135.62B	177.00A	5.66	0.0001
LDH (U/L)	604.47	616.89	7.92	0.2824
γ-GT (U/L)	13.05	15.43	1.24	0.1923
CHE (U/L)	78.01	79.58	4.45	0.8062

GLU, glucose; BUN, blood urea nitrogen; LDH, lactate dehydrogenase; CRE, creatinine; ALT, alanine aminotransferase; ALP, alkaline phosphatase; AST, aspartate aminotransferase; γ-GT, γ-glutamyl aminotransferase; CHE, cholinesterase.

### 3.4 Selenium content in blood and milk

Table 5 shows that NSe significantly increased the selenium content of milk by 9.96% (*p* < 0.05) compared to that of the CON group but did not affect the selenium content of the blood (*p* > 0.05).

**Table 5:** Effects of nanoselenium on the selenium content in the serum and milk of dairy cows during the peak lactation period.

Selenium content (μg/L)	CON	NSe	SEM	*p*-value
Blood Selenium	48.00	49.98	1.33	0.3167
Milk Selenium	19.58b	21.53a	0.27	0.0005

### 3.5 Milk metabolites

**Figure 1:**
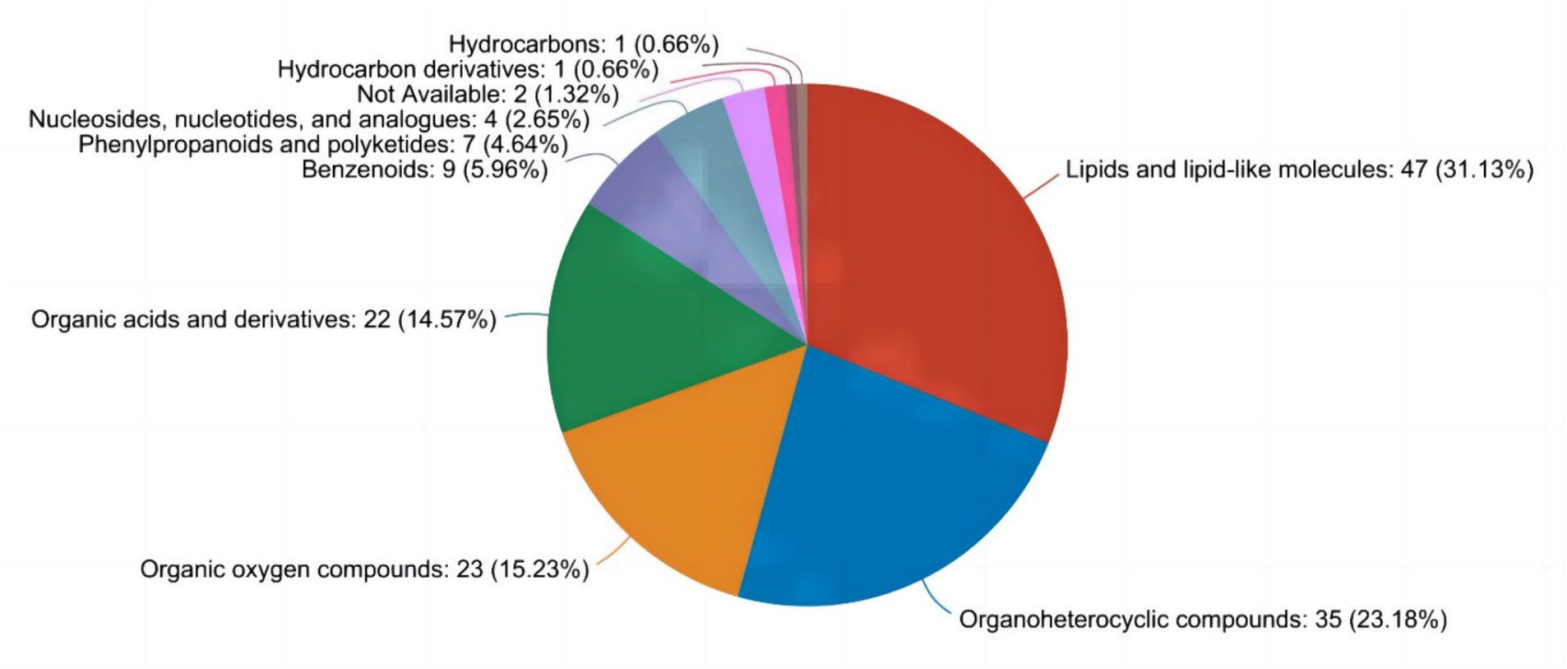
Analysis of metabolite species.

As illustrated in Figure 1, through the nontargeted metabolomics analysis of milk samples, a total of nine distinct types of metabolites were identified. Among these, organic heterocyclic compounds (24.14%), lipid and lipid molecules (33.10%), and organic acids and their derivatives (17.24%) comprised the top three categories of compounds.

**Figure 2:**
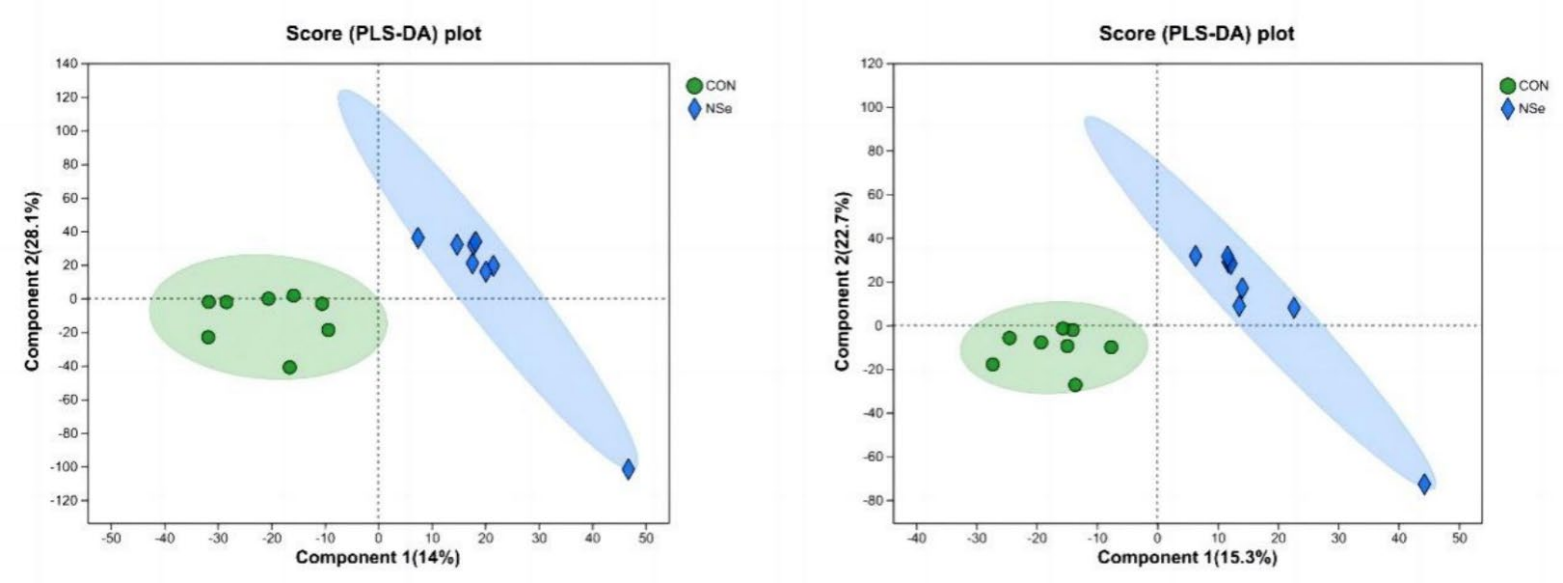
PLS-DA analysis (cationic model on the left, anionic model on the right); CON is the sodium selenite group, and NSe is the nanoselenium group.

The PLS-DA score chart (Figure 2) showed that the milk metabolites in both groups could be significantly separated. In the positive ion mode, 14% of the variance was explained by the principal component, and 28.1% of the variance was explained by the second principal component. In the negative ion mode, the first principal component explained 15.3%, and the second principal component explained 22.7%. All the samples were within the 95% confidence intervals, indicating that there was little difference in the number of samples in each group among the groups, and the projection area of the NSe group was significantly different from that of the CON group.

**Figure 3:**
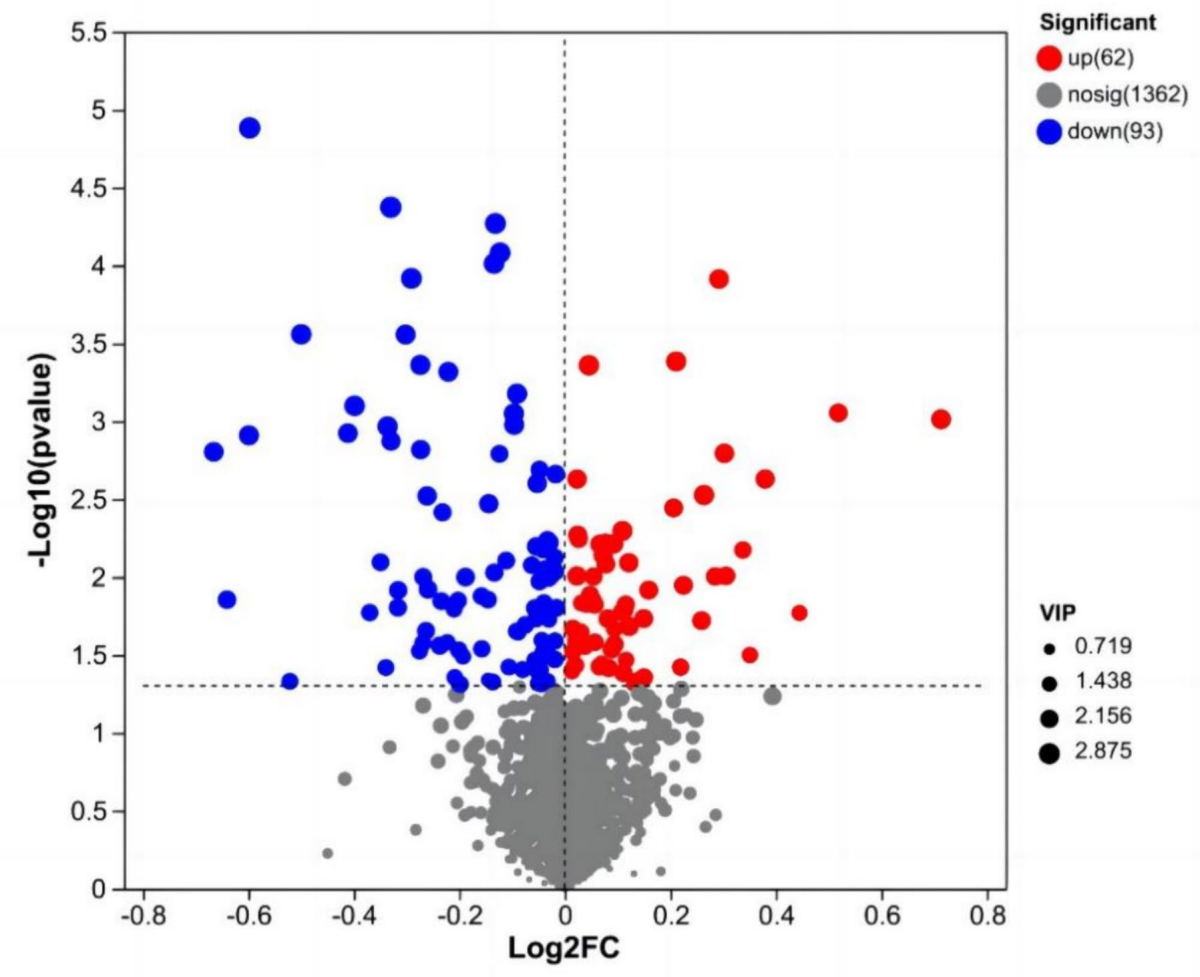
Volcano map of milk metabolites in the CON group and NSe group. Each dot represents a metabolite, with blue indicating downregulation and red indicating upregulation; VIP is the weight of the variable.

After data preprocessing, a student t-test analysis was carried out, and a volcano plot was generated according to the *p* values and FC values, as shown in Figure 3. A total of 155 differentially abundant metabolites were evaluated using the screening parameters FC > 1 or FC < 1, VIP > 1, and *p* < 0.05. Among these metabolites, 62 were downregulated, while 93 were upregulated. The results of some differentially abundant metabolites are shown in Table 6.

**Table 6:** Positive and negative ion mode statistical table for differentially abundant metabolite identification.

Metabolite	Retention time/min	Detected m/z	VIP	Difference multiple/FC	*p*-value	Tendency
Cetylmannoside	6.944	446.347	1.764	2.266	0.0094	Up
Allantoin	0.705	157.036	2.330	1.466	0.0004	Up
Undecylenic acid	6.798	367.285	2.107	15.065	0.0031	Up
3-hydroxypentadecanoic acid	6.542	257.212	2.131	4.835	0.0026	Up
16-Hydroxyhexadecanoic acid	6.630	271.227	1.569	2.967	0.0477	Up
Threonic acid	0.658	135.029	1.909	1.583	0.0087	Up
Coumarin	7.942	147.044	1.947	1.016	0.0099	Up
4-Decan-4-Ylbenzenesulfonic acid	6.502	297.153	1.740	1.011	0.0214	Up
Galactaric acid	2.834	252.072	2.249	0.363	0.0002	Down
CDP-glucose	0.779	566.077	1.500	0.115	0.0489	Down
5-Methoxy tryptophan	3.374	199.086	2.081	0.144	0.0010	Down
N6-Succinyl Adenosine	3.463	382.100	2.466	0.362	0.0001	Down
Riboflavin	4.468	375.130	2.248	0.426	0.0011	Down
Uric acid	0.816	335.049	2.109	0.020	0.0028	Down
Indolylacryloylglycine	5.943	243.077	1.799	0.419	0.0237	Down
Pyrimidopurinone	4.730	338.065	1.687	0.074	0.0315	Down
1-Methyladenosine	3.420	326.110	1.581	0.128	0.0386	Down
Succinic acid	1.488	117.018	1.955	0.978	0.0058	Down
Oxoglutaric acid	0.816	145.013	1.654	0.973	0.0268	Down
Mesaconic acid	1.263	129.018	1.798	0.989	0.0158	Down
Malic acid	0.801	133.013	1.778	0.973	0.0147	Down

FC means fold change, FC > 1 indicates that the content of the metabolite in the milk of the NSe group was greater than that in the CON group; FC < 1 indicates that the content of the metabolite in the milk of the NSe group was lower than that in the CON group. VIP value: Variable importance in projection between treatments from PLS-DA.

**Figure 4:**
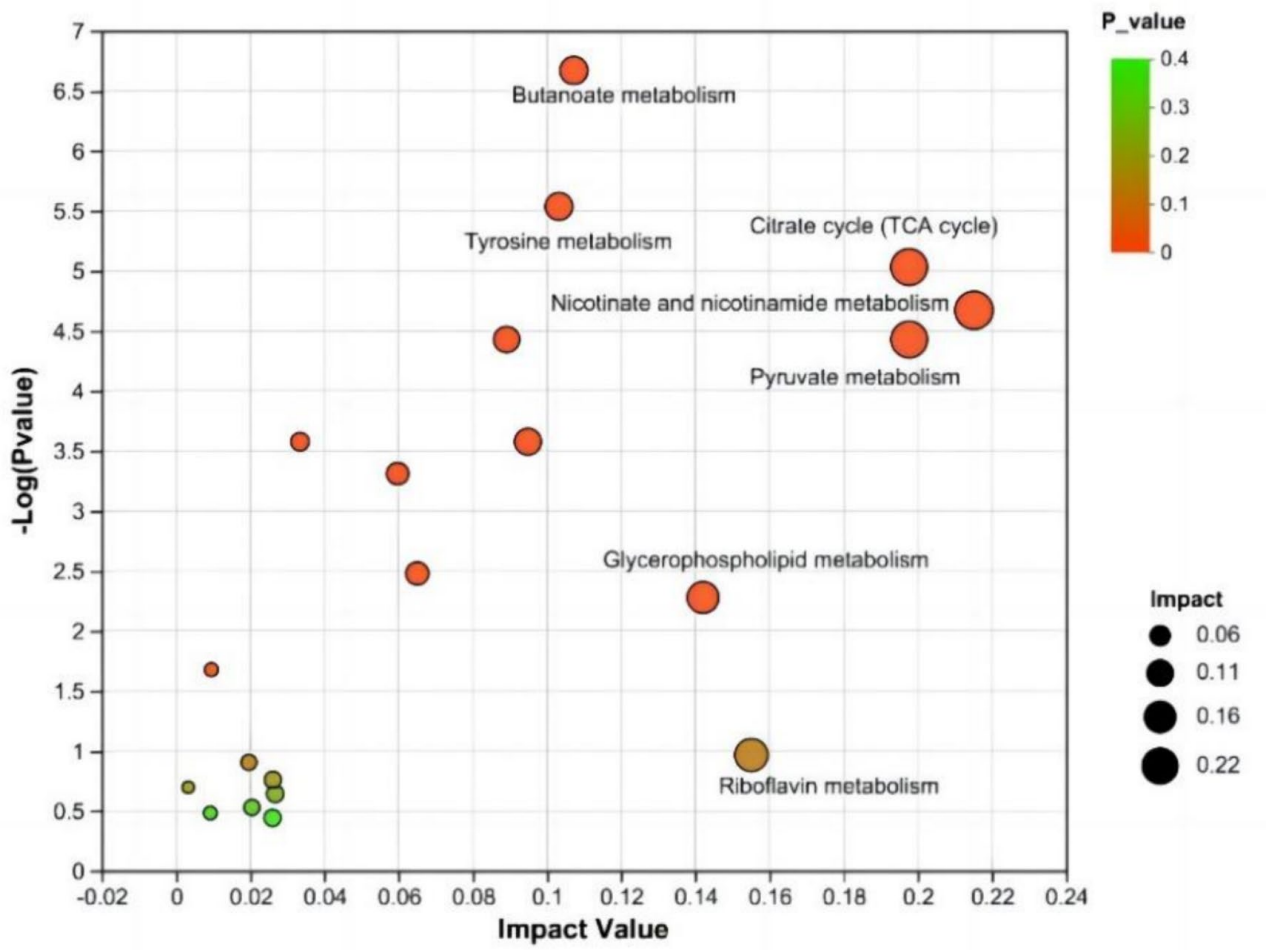
KEGG topology analysis. Each bubble denotes a KEGG pathway. The magnitude of the impact value, which illustrates the relative relevance of metabolites in the pathway, is represented along the horizontal axis. The significant enrichment of metabolites involved in the pathway is denoted along the vertical axis by -log10 (*p* value). Bubble size denotes impact value. The greater the bubble is, the more significant the path.

An enrichment analysis of the metabolic pathways associated with the differentially abundant metabolites between the CON and NSe groups was conducted. The findings demonstrated the involvement of 31 metabolic pathways involving 155 identified differentially abundant metabolites. Metabolic pathways affected by differentially abundant metabolites were screened using an impact value greater than 0.1. The main metabolic pathways involved were the metabolism of niacin and niacinamide, pyruvate, citrate cycle (TCA cycle), glycerophospholipid, riboflavin, butanoate and tyrosine. The results are shown in Figure 4 and Table 7.

**Table 7:** Differential enrichment of metabolite pathways between the CON group and NSe group.

Pathway	*p*-value	Impact value
Nicotinate and nicotinamide metabolism	0.0001	0.2152
Pyruvate metabolism	0.0001	0.1977
Citrate cycle (TCA cycle)	0.0001	0.1977
Riboflavin metabolism	0.1084	0.1551
Glycerophospholipid metabolism	0.0053	0.1421
Butanoate metabolism	2.1417	0.1073
Tyrosine metabolism	0.0001	0.1033

The impact value of pathways is calculated as the sum of the important measures of the matched metabolites, with the threshold above 0.10 are regarded as significant.

**Figure 5:**
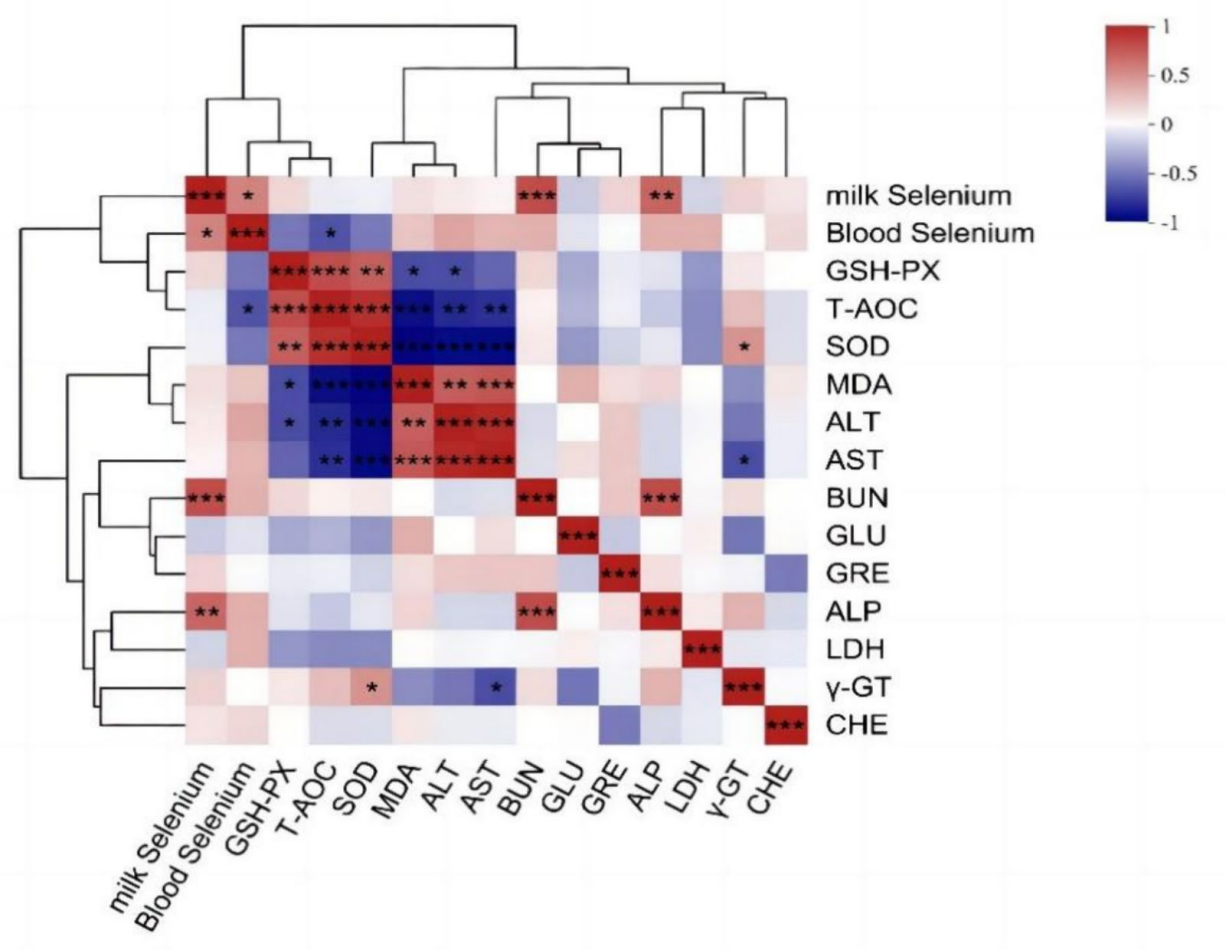
Correlation analysis results of the selenium content in blood milk and blood biochemical indices. The *R*-values of the correlation coefficients and the corresponding *p* values were obtained via calculations. The graph displays *R* values using various colors. *p* < 0.05 is represented by *, *p* < 0.01 is represented by **, and the legend on the right displays the color range for various *R* values. Red and blue represent positive and negative correlations, respectively. and color depth indicates the size of the data value. The image below is the same.

Pearson correlation analysis was performed to examine the relationships among blood selenium content, milk selenium content, and blood biochemical indices. Figure 5 shows a statistically significant positive correlation between the serum and milk selenium levels (*p* < 0.05). Additionally, a statistically significant negative correlation between the serum selenium concentration and the blood T-AOC was also detected (*p* < 0.05). The selenium content in milk was positively correlated with BUN and ALP (*p* < 0.01), and γ-GT was negatively correlated with AST (*p* < 0.05) and positively correlated with SOD (*p* < 0.05).

**Figure 6:**
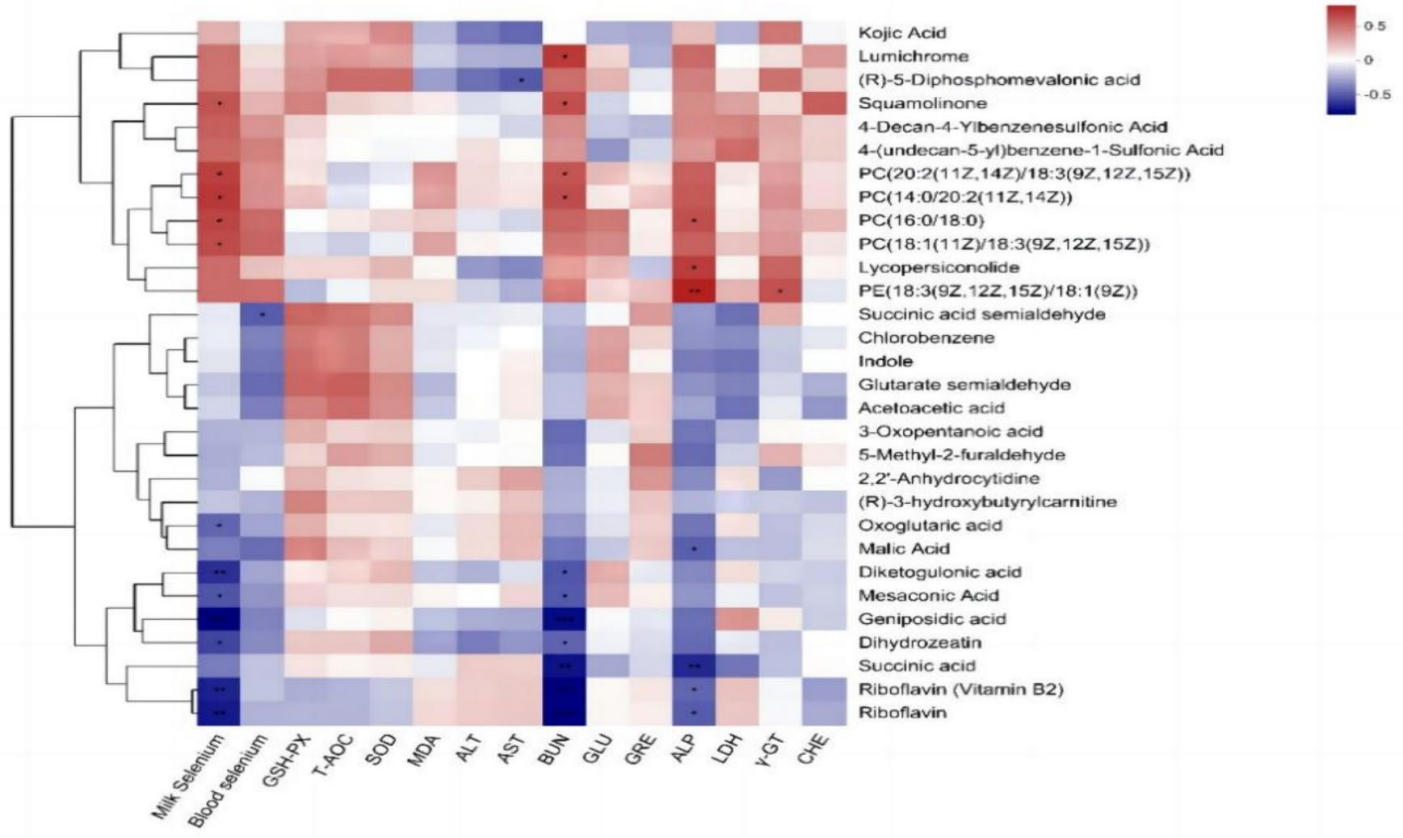
Pearson correlation analysis of the differentially abundant metabolites in milk with the blood selenium content, milk selenium content, and blood biochemical indices of dairy cows.

The Pearson correlation analysis of milk metabolites with blood selenium content, milk selenium content, and blood biochemical indices (Figure 6) revealed that the milk selenium content was significantly positively correlated with the milk metabolites squamolinone, PC [20:2 (11Z, 14Z)/18:3 (9Z, 12Z, 15Z)], PC [14:0/20:2 (11Z, 14Z)], PC (16:0/18:0) and PC [18:1 (11Z)/18:3 (9Z, 12Z, 15Z)] and was extremely significantly negatively correlated with riboflavin (vitamin B_2_), riboflavin, diketogulonic acid and geniposidic acid (*p* < 0.01) but significantly negatively correlated with oxoglutaric acid, mesoconic acid and dihydrozeatin (*p* < 0.05). The blood selenium content was significantly negatively correlated with the amount of succinic acid semialdehyde among the milk metabolites (*p* < 0.05). AST was negatively correlated with (R)-5-diphosphomevalonic acid (*p* < 0.05). BUN showed a significant positive correlation with lumichrome, squamolinone, PC (20:2 (11Z, 14Z)/18:3 (9Z, 12Z, 15Z), and PC [14:0/20:2 (11Z, 14Z)] (*p* < 0.05) and a highly significant negative correlation with riboflavin (vitamin B_2_), riboflavin, succinic acid, and geniposidic acid (*p* < 0.01). Additionally, BUN was significantly negatively correlated with diketogulonic acid, mesaconic acid, and dihydrozeatin (*p* < 0.05). ALP showed a highly significant positive correlation with PE [18:3 (9Z, 12Z, 15Z)/18:1 (9Z)] (*p* < 0.01), a significant positive correlation with the concentrations of lumichrome and squamolinone (*p* < 0.05), and a significant negative correlation with riboflavin, riboflavin (vitamin B_2_), and malic acid (*p* < 0.05). A positive correlation was observed between γ-GT and PE [18:3 (9Z, 12Z, 15Z)/18:1 (9Z)] (*p* < 0.05).

## 4 Discussion

Both excessive and insufficient selenium in animal diets can reduce animal performance (33, 34). According to the Nutrient Requirements of Dairy Cows (30, 35), the amount of selenium in diets ranges from approximately 0.3 mg/kg DM to 3 mg/kg DM. The amount of added selenium in this experiment was approximately 0.75 mg/kg DM. According to the results of the evaluation of clinical indications of selenium poisoning (Annex 1 for evaluation criteria) and the blood selenium content of dairy cows, there were no abnormal indications observed during the whole experiment, indicating that the selenium content in the diets was within an acceptable range.

Different types of selenium supplements have diverse effects on the performance of dairy cows. As indicated in Table 2, DMI is not affected by selenium sources, which is consistent with the research results of Sun et al. (36) and Juniper et al. (37). Furthermore, it was also confirmed that supplementation of selenium source seemed to have no effect on DMI of dairy cows, beef cattle, calves, and lambs (38). However, the milk production of dairy cows during the middle and late phases of lactation was found to be enhanced through the administration of dietary supplements containing selenium yeast (SY) and hydroxy-selenomethionine (OH-SeMet) (39). The increase in milk production in cows may or may not be directly related to the positive effects of selenium supplements (39, 40), since it is influenced by several factors, including nutritional composition, lactation status, rumen function, and overall health. However, in this experiment, on the basis of no significant change in DMI, the increase in milk yield of dairy cows was very close to the increase in feed efficiency. Therefore, it is reasonable to think that there is a certain correlation between milk yield and feed efficiency. Similar results have been reported by Wang et al. (41) that milk yields increased by selenium-yeast supplementation without change in DMI but improve the rumen fermentation. Dairy cows have a strong metabolism and high production intensity when they are at the peak of the lactation period. Which means that cows can obtain more nutrients from feed and use them more efficiently (42), which provides the material basis for the improvement of milk yield. On the other hand, selenium supplementation can strengthen the distribution of breast capillaries, and the breast vascular area tends to increase (43), which provides organizational conditions for the improvement of milk production. In addition, nanoselenium also has a unique size effect (nanoscale), making it more absorbable and more bioavailable in the small intestine than inorganic selenium (41, 44). This also explains the increase in the milk selenium content; that is, the conversion of feed selenium to milk selenium increased. In terms of milk composition, nanoselenium supplementation had no significant effects on milk fat, protein, lactose, or ash, which was in line with the results of earlier studies (41). This indicates that selenium has little effect on the structure and synthesis of mammary gland acinar cells (45), but this finding needs to be confirmed by further studies.

Glutathione peroxidase (GSH-Px) is an important indicator of the antioxidant capacity of animals. It can protect the structure and function of cell membranes by specifically catalyzing hydrogen peroxide reduction through reducing GSH (46), and selenium is its active component. Hence, selenium supplementation in the diet of dairy cows could theoretically enhance the activity of blood GSH-Px, ultimately enhancing their antioxidant capacity. However, the impacts of selenium supplementation on blood GSH-Px were reported to be different. Studies on fattening pigs, broilers, and dairy cows have shown that adding appropriate amounts of selenium can improve GSH-Px activity in serum and that the effect of organic selenium is greater than that of inorganic selenium (47–49). However, some investigations have demonstrated that supplementation with sodium selenite and selenium yeast in dairy cows has no significant effect on blood glutathione peroxidase (GSH-Px) (50, 51). These results are in line with the results of this investigation, which showed no significant changes in serum SOD, T-AOC, or MDA levels. T-AOC, SOD, and MDA are also important parameters that reflect the antioxidant status of animals. Body resistance to oxidative stress can be measured by the T-AOC. SOD is a significant indicator of antioxidant capacity in animals. It functions by eliminating free radicals, defending against the harmful effects of oxygen free radicals, and promptly repairing damaged cells. MDA is an important product of the peroxidation of membrane lipids and serves as an indicator of tissue damage caused by peroxidation. Selenium supplementation does not necessarily improve the antioxidant level of animals, and studies have shown that the antioxidant capacity of the body can even be reduced with selenium supplementation (52). The impact of selenium on the body’s antioxidant capacity depends on various factors, such as the time and amount of selenium supplementation, the lactation stage, nutrient supply, stress, and genetics.

Blood biochemical indices are important indicators reflecting the physiological metabolism and health status of animals (53). When the body lacks selenium, immune suppression occurs. In contrast, excessive selenium supplementation in the diet will lead to selenium poisoning in dairy cows and even cause liver and kidney necrosis, which are often reflected in changes in blood biochemical parameters (54, 55). At present, it has been found that appropriate supplementation of selenium in the diet can enhance the immune function of dairy cows, promote the production of immunoglobulin, increase antibody titres, and reduce diseases (56). The results of this trial demonstrated that NSe significantly increased the levels of BUN and ALP in the blood. But had minimal impact on other biochemical indicators, which were consistent with previous studies (4, 57). BUN, an amino acid and protein metabolite, can serve as an indicator of the equilibrium of protein and amino acid metabolism within an animal (58). BUN of ruminants is mainly derived from ammonia produced by rumen degradable protein (RDP) and the metabolites of rumen undegradable protein (UDP) that entering the stomach and small intestine. BUN is sent to the liver through the portal vein to synthesize urea, which excreted by the kidney or return to the rumen through saliva and rumen epithelium. The level of BUN is affected by many factors such as dietary crude protein (CP), RDP, sampling time, renal excretion and renal reabsorption. Studies have shown that the BUN content in cattle first increases after feeding and then decreases to premeal levels 8 h after feeding (59). Blood collection in this study was conducted 1 h before the morning feeding when the BUN level was close to the lowest level in a day and within the normal range [usually 4–16 mg/dL (60) or 1.4–5.7 mmol/L], indicating a sufficient protein in the diet. Since both groups had the similar serum creatinine (CRE) levels and BUN/CRE ratio was within the normal range (Usually <20), a normal renal excretion function could be expected. Therefore, the increase of BUN in the NSe group is more likely be related to enhancement of nitrogen in the digestive tract or the reabsorption of renal, both of which could be good for protein utilization. A previous study has proved that the BUN of high-yielding cows is higher than that of low-yielding cows (61). On the other hand, the activity of alkaline phosphatase (ALP) in the blood reflects the metabolic activity and functional status of animal tissues and organs (62). ALP is usually elevated when animals develop liver and bone diseases or tumors; however, it is sometimes also elevated because of physiological reasons, such as in young animals, pregnant and lactating females, or the intake of high-fat diets (63). It has been reported that the damage caused by selenium poisoning mainly occurs in the liver (64), But based on the normal concentrations of AST and ALT, the indicators of hepatocyte injury and severity (57), it was speculated that it may be related to high milk production or subclinical mastitis, rather than abnormal liver functions. The reason is that ALP is closely associated with lowering blood calcium and promoting bone deposition. When calcium mobilization is excessive in the body, the compensatory proliferation of osteoblasts increases the level and activity of serum ALP to maintain the stability of the calcium content in the body to ensure calcium deposition in the body (65). When the milk yield increased in cows of the NSe group, the animals mobilized and consumed more calcium, and the increased blood ALP content allowed the cows to maintain normal calcium deposition.

After selenium is absorbed in the intestinal tract of an animal, it can enter the blood or be transferred into the milk, so the contents of blood selenium and milk selenium can reflect the use status of selenium in cows. The blood selenium content is often related to the binding ability of selenium, and studies have shown that adding organic selenium to the diet has a greater effect on increasing the blood selenium concentration than does adding inorganic selenium (66, 67). This could be due to the increased binding ability of organic selenium to α- and β-globulin, LDL (low-density lipoprotein), VLDL (very low-density lipoprotein), and albumin in the blood (68). Nanoselenium may also have a higher binding degree than sodium selenite, because the increase of blood selenium (like glutathione), milk selenium as well as the stimulation of selenoprotein gene expression in the mammary glands of dairy cows by nanoselenium supplementing were also reported (23). The positive correlation between selenium in blood and in milk may be related to the binding of selenium with protein, and selenium is considered first bound to amino acids or proteins in the blood (69) and subsequently absorbed and integrated by the mammary gland to synthesize milk proteins (70). However, the blood selenium level in the NSe group in this trial was only numerically higher than the CON group, but not statistically significant (*p* > 0.05), only milk selenium levels increased significantly (*p* < 0.05). Due to the small size of nanoselenium particles (nanoscale), further tests are needed to confirm whether nanoselenium does not completely bind to proteins in the blood, but is rapidly transferred from breast capillaries to the milk.

As the most common source of nutrients for human beings, milk products not only provide people with basic nutrients but also have a higher concentration of small molecule metabolites, such as organic acids, fatty acids, amino acids, nucleotides, and bioactive peptides (71). Therefore, the milk metabolites of Holstein cows at the peak lactation period were analyzed via nontargeted metabolomics (LC–MS) in this study. The findings demonstrated that NSe significantly increased the contents of threonic acid, cetylmannoside, undecylenoic acid, 3-hydroxypentadecanoic acid, 16-hydroxypentadecanoic acid, etc. Threonic acid is an active metabolite of ascorbic acid catabolism, also can be derived from glycated proteins (72), and was proved to be beneficial for the absorption of calcium and for supplementing ascorbic acid (73). This helps to ensure high milk production. 3-hydroxypentadecanoic acid and 16-hydroxypentadecanoic acid both are hydroxyl fatty acids, which were found to be present in the milk (74). Cetylmannoside activates the complement system in the form of liposomes (75). While undecylenoic acid was proved to have antifungal activity (76). In this study, the levels of galactaric acid, mesaconic acid and CDP-glucose etc. in the NSe group milk were decreased. Galactaric acid is a product of microbial decomposition of pectin, which helps to maintain the stability of foods (such as yogurt) (77, 78). Mesaconic acid is the degradation product of citric acid in milk (79). CDP-glucose participates in the synthesis of lactose (80). Although the milk glucose concentration of all mammals (except humans) is low (0.1–0.3 mmol/L), the milk glucose concentration can still reflect the glucose concentration in mammary cells. When the efficiency of lactose synthesis in mammary cells decreases, the milk glucose concentration decreases (81).

In metabolic pathways where differentially abundant metabolites are concentrated, niacin and niacinamide, pyruvate and the citrate cycle (TCA cycle) are mainly related to metabolites such as mesaconic acid, succinic acid, oxoglutaric acid, and malic acid in milk. Riboflavin is mainly involved in riboflavin metabolism. The glycerophospholipid metabolic pathway is mainly related to lipid metabolism, such as PE [18:1 (9Z)/18:3 (9Z, 12Z, 15Z)] and PC [18:1 (11Z)/18:3 (9Z, 12Z, 15Z)], and metabolism of butanoate and tyrosine pathways are mainly related to metabolites such as succinic acid semialdehyde, acetoacetic acid, succinic acid and maleic acid.

## 5 Conclusion

Under short-term experimental conditions, NSe significantly increased the milk yield, milk selenium content, and feed conversion rate (*p* < 0.05). However, no significant impact was observed for other milk components (*p* > 0.05). NSe significantly increased blood BUN and ALP levels (*p* < 0.05) but did not have a significant impact on blood T-AOC, GSH-Px, SOD, or MDA levels or on blood selenium content (*p* > 0.05). In general, the efficiency of nanoselenium converting from feed selenium to milk selenium is greater than that of sodium selenite, and nanoselenium has certain regulatory effects on blood biochemical indices, milk metabolites, and related metabolic pathways. It has potential for application in cow production.

## Data availability statement

The original contributions presented in the study are included in the article/supplementary material, further inquiries can be directed to the corresponding author.

## Ethics statement

The animal study was approved by all animals used in this study were reviewed and approved by the Ethics Committee of Inner Mongolia Minzu University. (Ethics Review Number: 2022092016001). The study was conducted in accordance with the local legislation and institutional requirements.

## Author contributions

MX: Writing – original draft. YaW: Writing – original draft, Writing – review & editing. MW: Writing – original draft, Writing – review & editing. WP: Writing – original draft. YuW: Writing – original draft. RZ: Writing – original draft. YZ: Writing – original draft. JJ: Writing – original draft. CD: Writing – original draft. LD: Writing – original draft. MB: Writing – original draft.

## Footnotes

^1^ https://www.kegg.jp/kegg/pathway.html
